# Finding a Potential Dipeptidyl Peptidase-4 (DPP-4) Inhibitor for Type-2 Diabetes Treatment Based on Molecular Docking, Pharmacophore Generation, and Molecular Dynamics Simulation

**DOI:** 10.3390/ijms17060920

**Published:** 2016-06-13

**Authors:** Harika Meduru, Yeng-Tseng Wang, Jeffrey J. P. Tsai, Yu-Ching Chen

**Affiliations:** 1 Department of Bioinformatics and Medical Engineering, Asia University, 500, Lioufeng Rd., Wufeng, Taichung 41354, Taiwan; harikameduri.bioinfo@gmail.com (M.H.); president@asia.edu.tw (J.J.P.T.); 2 Department of Biochemistry, Kaohsiung Medical University, 100, Shih-Chuan 1st Road, Kaohsiung 80708, Taiwan; c00jsw00@kmu.edu.tw

**Keywords:** incretin, GLP-1, GIP, DPP-4 inhibitors, type-2 diabetes, CDOCKER, pharmacophore generation, molecular dynamics simulation

## Abstract

Dipeptidyl peptidase-4 (DPP-4) is the vital enzyme that is responsible for inactivating intestinal peptides glucagon like peptide-1 (GLP-1) and Gastric inhibitory polypeptide (GIP), which stimulates a decline in blood glucose levels. The aim of this study was to explore the inhibition activity of small molecule inhibitors to DPP-4 following a computational strategy based on docking studies and molecular dynamics simulations. The thorough docking protocol we applied allowed us to derive good correlation parameters between the predicted binding affinities (pK_i_) of the DPP-4 inhibitors and the experimental activity values (pIC_50_). Based on molecular docking receptor-ligand interactions, pharmacophore generation was carried out in order to identify the binding modes of structurally diverse compounds in the receptor active site. Consideration of the permanence and flexibility of DPP-4 inhibitor complexes by means of molecular dynamics (MD) simulation specified that the inhibitors maintained the binding mode observed in the docking study. The present study helps generate new information for further structural optimization and can influence the development of new DPP-4 inhibitors discoveries in the treatment of type-2 diabetes.

## 1. Introduction 

Worldwide 314 million individuals are suffering from (metabolic disorder) type-2 diabetes mellitus, which has been classified as a disease of glucose overproduction by tissues lacking enough insulin production [1]. Insulin resistance is one of the major associated conditions for most patients with the disorder, and is generally present in people suffering with obesity [2]. The infirmity of insulin secretion and insulin action on glucose uptake in muscles leads to type-2 diabetes mellitus [3]. Type-2 diabetes is radically increasing throughout the world and, according to the estimates of the International Diabetes Federation (IDF), by the year 2030 the total number of people with diabetes is estimated to reach 552 million [4]. 

**Figure 1:**
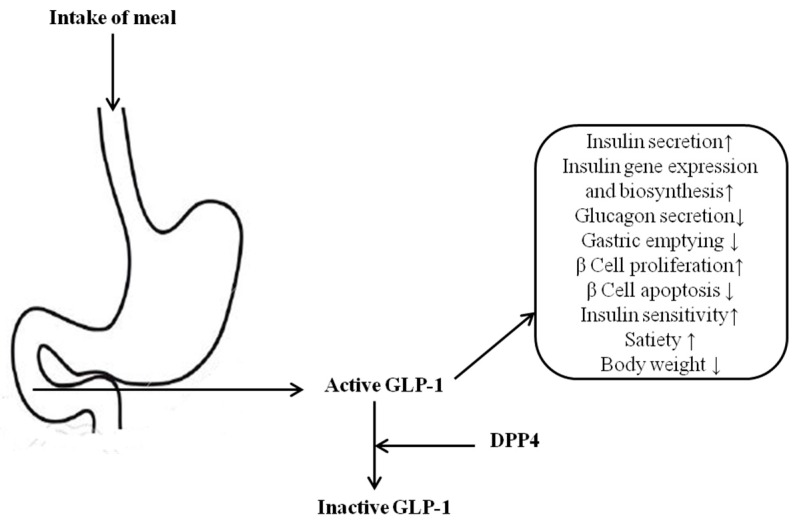
Glucagon-like peptide 1 (GLP-1) activation effects by meal intake, and its metabolism through dipeptidyl peptidase-4 (DPP-4) [6].

The function of glucagon like peptide-1 (GLP-1) and its potential role in treating type-2 diabetes was first reported in late 1990s [5]. Glucagon-like peptide-1 (GLP-1) and glucose-dependent insulinotropic peptide (GIP) are intestinally derived incretin hormones, which physiologically arouse insulin emissions from β-cells postprandially (Figure 1) [6]. GLP-1 has multiple advantageous effects on beta-cell chore and mass by stimulating beta-cell development from precursor cells and inhibiting beta-cell apoptosis [5,7,8].The reduction of blood glucose and hemoglobin A1c levels in type-2 diabetes patients as triggered by the continuous infusion of GLP-1 is a significant result. However, the active GLP-1 and GIP are degraded to inactive metabolites by the enzyme called dipeptidyl peptidase-4 (DPP-4) [7]. Due to the extremely short biological half-life, treatment with native GLP-1 for type-2 diabetes is not adequate.

After the ingestion of a meal, ileal L-cells produce active GLP-1 and influence its action through: (1) stimulation of insulin liberation; (2) glucagon release inhibition; and (3) slowing down the gastric emptying process [9]. GLP-1 analogues and DPP-4 inhibitors belong to one of the incretin-based therapies for type-2 diabetes [10,11]. GLP-1 analogues (liraglutide and exenatide) are not suitable for oral delivery because of their biochemical nature, and must be injected subcutaneously on a daily basis. The usage of GLP-1 analogues was limited to agents because of its requirement for injection, whereas competitive DPP-4 inhibitors, which are chemically plagiaristic, are administered orally [9,10,12].

**Figure 2:**
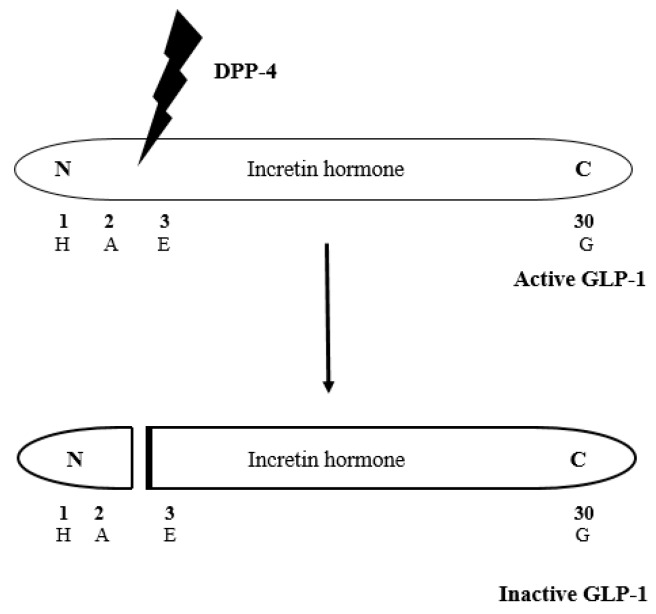
DPP-4 cleaves the incretin hormones GLP-1 and glucose-dependent insulinotropic peptide (GIP) in their second position where proline or alanine is present [15,16].

Human DPP-4 is signified as a single polypeptide chain of 766 amino acids, which consists of a N-terminal cytoplasmic domain, a trans-membrane domain, a β-propeller domain, and a α/β-hydrolase domain [11,13]. DPP-4 has different types of known substrates and contains growth factors like chemokines, neuropeptides, and vasoactive peptides [14]. Reports show that DPP-4 has a significant function in glucose metabolism; furthermore, it is responsible for degrading incretins like GLP-1 and GIP through cleaving their amino acids which have proline or alanine in the second position (Figure 2) [15,16]. To overcome this predicament, the inhibition of the enzyme DPP-4, which prevents the inactivation of GLP-1, thus intensifies and prolongs the action of the incretin hormone.

A computational approach involving molecular docking refined with molecular dynamics simulations as well as a pharmacophore analysis could pave the way for new insights on potential DPP-4 inhibitors for type-2 diabetes treatments, as successfully described in the literature about other case studies [17,18,19,20]. Finding small molecular inhibitors through computer-aided technologies has been successful for disease diagnosis and therapeutic interventions in previous research [21,22,23,24,25]. With this in mind, *in silico* studies have been deployed for the identification of DPP-4 inhibitors. In the present study, we have reported molecular interaction studies including molecular docking and molecular dynamics to order to understand the stability of the complex. Furthermore, pharmacophore generation was used in order to recognize how structurally diverse compounds bind in the specific receptor site. The, integration of these methods might help to develop a potential DPP-4 inhibitor for treating type-2 diabetes. 

## 2. Results and Discussion

### 2.1. Molecular Docking

In the present study, we collected structurally diverse small molecules, such as aminopiperidine fused imidazoles, thiazolopyrimidine derivatives, and quinolin-fused imidazoles (Figure S1), from the literature [26,27,28]. Molecular docking was performed on these inhibitors in the effort to study the binding modes and to reveal the most essential residues involved in binding interactions. The following amino acids were involved in H-bond interactions: Arg125, Glu205, Glu206, Ser209, Arg358, Tyr547, Tyr631, Tyr662, Tyr666, and Asn710, (these amino acids are active site pocket residues of 2P8S) [29].

**Figure 3:**
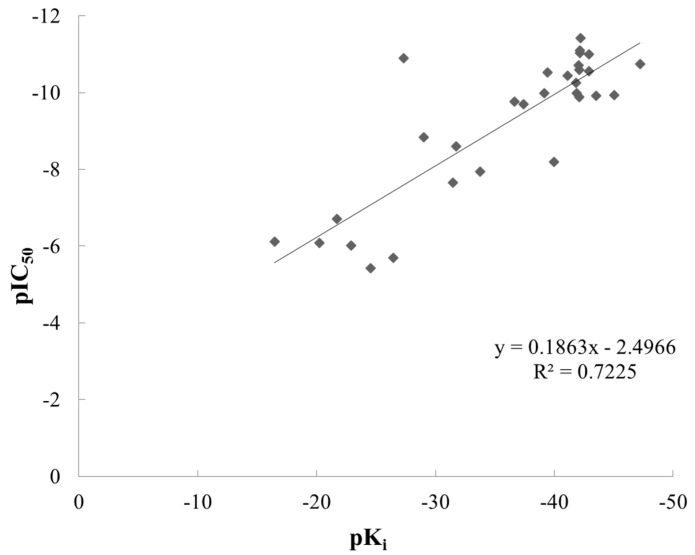
Relationship between the experimental pIC_50_ and the calculated pK_i_ of 31 inhibitors (*R*^2^ = 0.72).

A linear equation was developed for the predicted binding affinities (pK_i_) decision by using CDOCKER and experimental activity values (pIC_50_) (Figure 3). Between pIC_50_ and the pK_i_ of 31 diverse inhibitors, a linear correlation was obtained that yielded a good correlation coefficient (*R*^2^ = 0.72). Moreover, it can be observed that the 31 compounds are well strewn around the fitting line and without significant outliers. 

**Figure 4:**
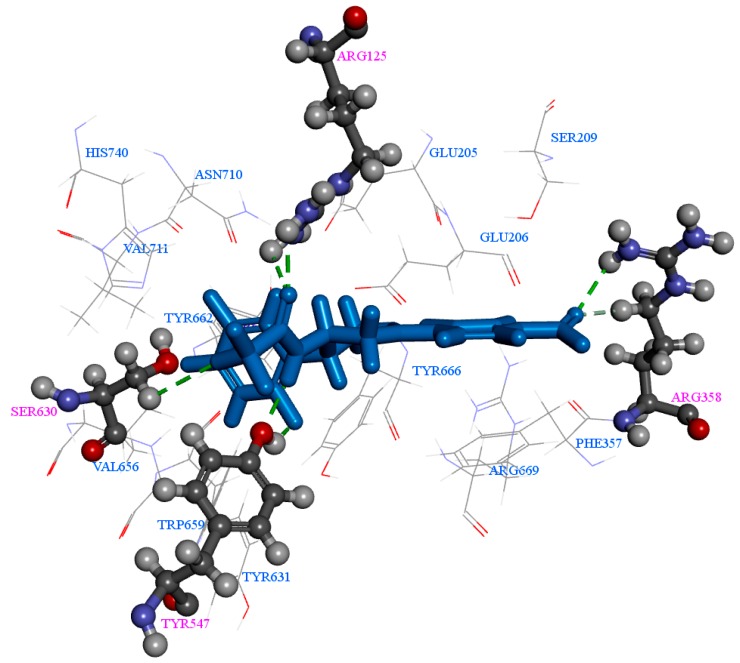
Molecular docking (CDOCKER) interactions of 2P8S_Comp71 with seven H-bonds. H-bond interactions are denoted as dotted lines. Active site amino acid residues Arg125, Arg358, Tyr547, and Ser630 are participating in H-bonding. There is one H-bond from Ser630 and two H-bonds from Arg125, Arg358, and Tyr547, respectively.

The top 10 compounds were selected from the 31 compounds for the present study by CDOCKER energy scores (Table 1) and whether they could bind with a 2P8S receptor to form more stable complexes than three existing drugs: sitagliptin (−39.43 kcal/mol), alogliptin (−25.64 kcal/mol), and vildagliptin (−5.64 kcal/mol). In this study Comp71 has the lowest CDOCKER energy score (−47.22 kcal/mol) with seven H-bonds (Table 1, Figure 4). 

### 2.2. Pharmacophore Generation

In a receptor site, a set of structural features in a molecule is recognized and is responsible for that molecule’s biological activity—this set of structural features is called a pharmacophore. The generated pharmacophore models based on receptor-ligand interactions have confirmed all substantial interactions in the compound-receptor interaction modes. The number of features, feature set, and selectivity score from pharmacophore generation is reported in Table 2. The ranking of pharmacophores are based on selectivity (arbitrary) scores—the higher the better. The top ten compounds with the highest arbitrary scores were chosen out of 31 compounds, and seven of them are all better than the existing drugs sitagliptin (5.63), vildagliptin (7.07), and alogliptin (8.19). 

**Figure 5:**
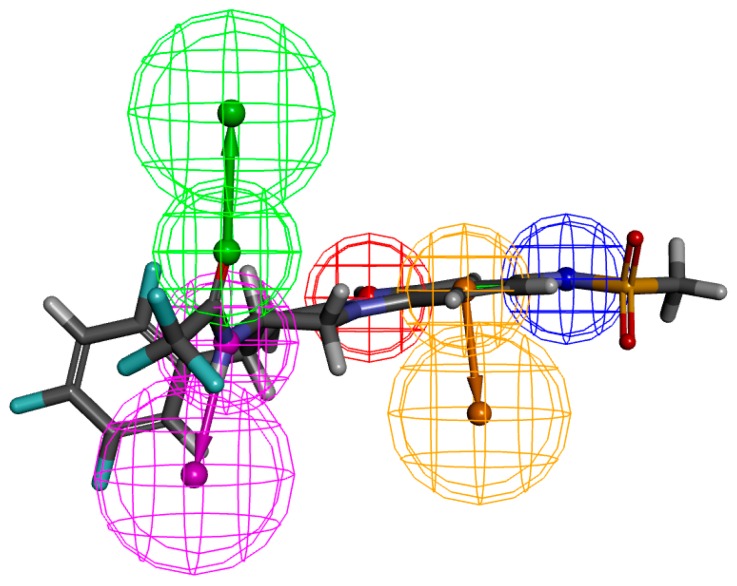
The result of pharmacophore features of 2P8S_Comp73 based on Receptor-Ligand Pharmacophore Generation. The hydrogen bond acceptor, hydrogen bond donor, positive ionizable feature, aromatic ring, and negative ionizable features are shown as green, magenta, red, orange, and blue, respectively.

In fact, Comp73 has highest selectivity score 11.72 and possesses five features: HB_ACCEPTOR (Hydrogen bond acceptor), HB_DONAR (Hydrogen bond donor), NEG_IONIZABLE (Negative ionizable feature), POS_IONIZABLE (Positive ionizable feature), and RING_AROMATIC (Aromatic ring). Our analysis identified oxygen, as specified as HB_ACCEPTORS (green spheres), are common pharmacophore features for all the compounds. Interactions between Comp73 and 2P8S can be explained by the recognition of POS_IONIZABLE features (red sphere), RING_AROMATIC features (orange sphere), NEG_IONIZABLE feature (blue sphere), and HB_DONOR (magenta sphere) features at nitrogen positions N12, the ring aromatic region, and N5 and N28 of Comp73, respectively (Figure 5).

### 2.3. Molecular Dynamics Simulation 

The top 10 high binding compounds from each CDOCKER and pharmacophore simulations were considered, out of which five compounds (Comp65, Comp71, Comp72, Comp73, and Comp74) were the same in both simulations (Table 1 and Table 2). As such, a total of 15 compounds and three existing inhibitors were further evaluated through MD simulations. Analysis of MD simulation results at different time points over the simulation trajectory confirm that H-bonds are formed and interrupt with protein side chains. The total number of intermolecular H-bonds at different time points for each ligand are specified in Table 3. For Comp70, the minimum intermolecular H-bonds with 2P8S at the active site region were seven to a maximum of 12 during the simulation trajectory. H-bonds were not constant and were disrupted during the simulation period; however, three stable H-bonds in Comp70 were formed with Glu205, Glu206, and Arg358. At the end of 1000 ps simulation, Ser209 and Ser630 were also significant residues in contributing to the 12 H-bonds. However, the H-bonds formed between residues Tyr631 and Asn710 with Comp70 were only at 200 ps in the simulation trajectory. The number of H-bonds varies from the CDOCKER and MD simulation because of diverse algorithms in each simulation. 

**Figure 6:**
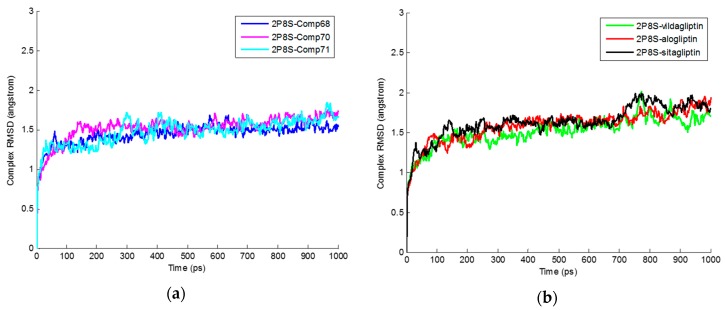
Root mean square deviations (RMSD) values of Protein ligand complexes all through molecular dynamics (MD) simulation at different time levels. (**a**) RMSD values of 2P8S_Comp68, 2P8S_Comp70, and 2P8S_Comp71 are present as blue, magenta, and cyan, respectively; (**b**) RMSD values of 2P8S_Vildagliptin, 2P8S_Alogliptin, and 2P8S_Sitagliptin are shown as green, red, and black, respectively.

**Figure 7:**
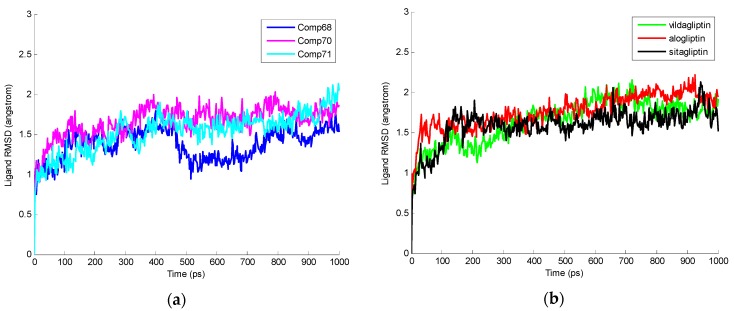
RMSD values of ligands through MD simulation at different time levels. (**a**) RMSD values of Comp68, Comp70, and Comp71 are present as blue, magenta, and cyan, respectively; (**b**) RMSD values of Vildagliptin, Alogliptin, and Sitagliptin are shown as green, red, and black, respectively.

Protein-ligand complexes and individual root mean square deviations (RMSDs) of ligands were obtained through MD simulation and are shown in Figure 6 and Figure 7. For each ligand the moderate RMSD value was calculated over the simulation trajectory. The average RMSD values of protein-ligand complexes and individual ligands are mentioned in Table 4. Similar RMSD values for individual ligands were observed and receptor-ligand complexes suggest the ligands can have stable interactions with their specific receptors. 

**Figure 8:**
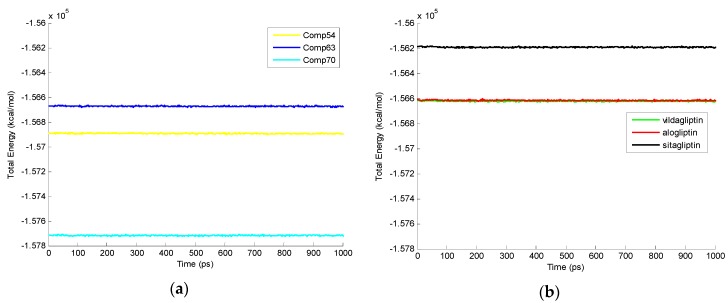
Total energies of protein-ligand complexes interaction during 1000 ps MD simulation. (**a**) Total energy of MD in complexes 2P8S_Comp54, 2P8S_Comp63, and 2P8S_Comp70 depicted as yellow, blue, and cyan plots, respectively; (**b**) Total energy of MD in complexes 2P8S_vildagliptin, 2P8S_alogliptin, and 2P8S_sitagliptin depicted as green, red, and black plots, respectively.

The total energies of 18 protein-ligand complexes through MD simulation listed in Table 3 with H-bonding and energies are also shown in Figure 8 at different time levels over 1000 ps simulation. In molecular dynamics simulation, Comp70 (−157,714.28 kcal/mol), Comp54 (−156,891.72 kcal/mol), and Comp63 (−156,672.61 kcal/mol) have the lowest total energies compared to the existing drugs vildagliptin (−156,620.70 kcal/mol), alogliptin (−156,613.52 kcal/mol), and sitagliptin (−156,192.52 kcal/mol). Among these three compounds in this study molecular dynamics results show that Comp70 with 12 H-bonding interaction during the MD simulation trajectory is an aminopiperidine fused imidazole and has the lowest total energy. 

**Figure 9:**
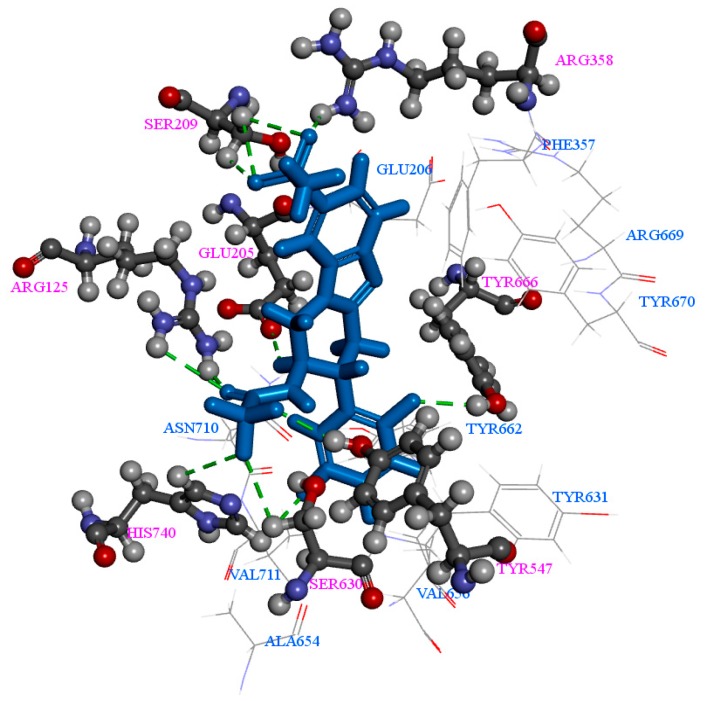
Intermolecular interactions of 2P8S_Comp70 during MD simulation. 12 H-bond collaborations are denoted as dotted lines. Amino acids displayed in this figure are all active site residues. The Arg125, Glu205, Ser209, Arg358, Tyr547, Ser630, Tyr666, and His740 participate in H-bonding; there are two H-bonds from Arg125, one H-bond from Glu205, three H-bonds from Ser209, one H-bond from Arg358, one H-bond from Tyr547, one H-bond from Ser630, one H-bond from Tyr666, and two H-bonds from His740.

Furthermore, Arg125, Glu205, Ser209, Arg358, Tyr547, Ser630, Tyr666, and His740 are the crucial amino acids (Figure 9) in formation of 12 H-bonding interactions with Comp70 during MD simulation. Amino acids Arg125, Arg358, Tyr547, and Ser630 are participating in both CDOCKER and MD simulations during the H-bond formation. Therefore, these significant residues might give guidance to develop more potent DPP-4 inhibitors. Comp70 has showed better simulation results than the existing drugs vildagliptin, alogliptin, and sitagliptin.

## 3. Methods and Materials

### 3.1. Small-Molecules Preparation

We collected 82 small molecule compounds to inhibit DPP-4 enzyme from the literature [26,27,28]. By using the equation Δ*G*_exp_ = −R*T*ln(IC_50_), IC_50_ values of inhibitors were converted to binding free energies (Δ*G*_exp_). Structures of inhibitors and biological activities (IC_50_) were shown in Figure S1. The compounds were drawn through Accelrys Draw v4.1 [30,31] to get SMILE formats, and simultaneously through Accelrys Discovery Studio v4.1, the SMILE formats were converted to .sdf files. Compounds were prepared using the “Prepare Ligand” protocol in Discovery Studio v4.1 with default parameters towards performing the CDOCKER simulation [32]. 

### 3.2. DPP-4 Model Preparation

In the present study DPP-4 was retrieved from RCSB PDB (entry code: 2P8S) with the cyclohexalamine inhibitor. 2P8S is a dimer of two chains, only the monomeric unit was used in the docking studies. The binding site (β-amino butyl amide moiety) for 2P8S was experimentally verified and the coordinates of the validated cyclohexalamine complex with 2P8S are available online [29]. The bounded cyclohexalamine inhibitor from the 2P8S structure was removed for docking experiments. Prepare Protein protocol performed tasks such as inserting missing atoms in incomplete residues, modeling missing loop regions, and removing waters from protein. The default parameter values were mostly kept the same in the “Prepare Protein” protocol from Discovery Studio v4.1 for DPP-4 model preparation.

### 3.3. Molecular Docking

Molecular docking of 82 selected DPP-4 inhibitors (from literature) to the 2P8S receptor was carried out in the present study by using CDOCKER with Discovery Studio v4.1. To perform flexible docking, for the small-molecules all torsion angles were set to be free. CDOCKER is a powerful CHARMm-based docking method that has been used to generate highly accurate docked poses. In this refinement application, the ligands were conceded to tilt around the rigid receptor [32]. In the CDOCKER simulation the following parameters were used: Top Hits-10; RandomConformations-10; Orientations to Refine-10; Force field-CHARMm; and Use Full Potential-False. For the ascertainment of potential correlations between experimental activities and predicted *K*_i_ values the best docked conformations of small-molecule inhibitors were selected as preliminary binding conformations.

### 3.4. Pharmacophore Generation

Receptor-ligand interactions are understood in detail with the advancement of pharmacophore generation. Through the pharmacophore model, the binding of structurally diverse ligands to common receptors active sites can be visualized [33]. In the present study we performed Receptor-Ligand Pharmacophore Generation with Discovery Studio v4.1 to know the interactions between protein and ligand. The Receptor-Ligand Pharmacophore Generation generates a set of selective pharmacophore models from a receptor-ligand complex. The model is generated from the features that correspond to the receptor-ligand interactions (CDOCKER interactions). The following ligand features types were considered: Hydrogen bond acceptor (HB_ACCEPTOR), Hydrogen bond donor (HB_DONOR), Hydrophobic feature (HYDROPHOBIC), Negative ionizable feature (NEG_IONIZABLE), Positive ionizable feature (POS_IONIZABLE), and Aromatic ring (RING_AROMATIC) [34]. The ranking of the top pharmacophore models was based on sensitivity and selectivity measurements. The selectivity of enumerated pharmacophore models is assessed with the Genetic Function Approximation (GFA) model. In Discovery Studio, the GFA model is implanted by the default settings of the GFA algorithm [35,36]. From a training set of 1544 pharmacophore models, the GFA model for selectivity of a pharmacophore is constructed. This set is used for searching a database of 5384 diverse, drug-like molecules and the logarithmic values of the number of database search hits are used as the targets (GFAscore). According to the following Equation (1), selectivity is derived from the GFAscore:

Selectivity = 11 – GFAscore
(1)

11 is a constant which ensures the selectivity score to result in positive values. 

### 3.5. Molecular Dynamics Simulations

To know the interaction strength and stability of the receptor-ligand complex molecular dynamics (MD) simulation studies were conceded. Discovery Studio v4.1 was utilized as a platform to perform MD simulations by means of Standard Dynamics Cascade. Prior to performing MD simulations, CHARMm polar hydrogen force field was applied to each of the protein-ligand complexes and solvation was set to explicit periodic boundary. The parameters for MD simulations were set with following conditions: Both steepest descent of energy minimization and steps of conjugate gradient minimization were kept to 300 in order to obtain constant and reasonable conformation of biomolecules. The system was heated from an initial temperature of 50 K to the target temperature of 300 K within 4 ps, and the equilibration steps were set to 1000. Moreover, the parameters of electrostatics were chosen as Particle Mesh Ewald (PME) for long-range electrostatic constrains and apply SHAKE Constraint was chosen as true, respectively. The total production time of 1000 ps simulations were performed with a type of run set as NVE (dynamics without temperature/pressure control). Default setting values were adopted for other parameters [37]. To analyze root mean square deviations (RMSDs) of protein-ligand complexes and ligands, we utilized the functions of Analyze Trajectory. After MD simulation, analyze trajectory functions were used to get RMSDs of protein-ligand complexes and ligands, total energies, as well as H-bonding interactions. All the computational procedures were performed on a dual-processor workstation (Intel(R)Xeon, 2.40 GHz) with a memory size of 16 GB.

## 4. Conclusions

In this study, we have explored structurally diverse compounds from the literature and performed molecular docking (CDOCKER) which revealed the importance of DPP-4 central β-amino butyl amide moiety for binding modes. Following this procedure, several selected compounds were submitted to pharmacophore generation and MD simulations. Notably, this analysis allowed us to identify DPP-4 inhibitors Comp68, Comp70, and Comp71 with effective selectivity scores and MD total energies. Moreover MD results of Comp70 showed the lowest total energy value with 12 H-bonding interactions and the ligand was stable according to its RMSD during MD simulation. Based on these computational methods, we have recognized that Comp70 might have the possibility to become an effective inhibitor for DPP-4, which belongs to the class of aminopiperidine-fused imidazoles. Computational analysis such as quantitative structure activity relationship (QSAR) and biological investigations are necessary in the further study. 

## Figures and Tables

**Table 1:** Calculated energies of CDOCKER and the number of H-bonds involved during molecular docking simulation.

Comp	CDOCKER Energy (kcal/mol)	Number of H-Bonds
71	−47.22	7
72	−45.05	7
64	−43.55	6
65	−42.93	7
74	−42.93	7
68	−42.20	7
69	−42.16	7
73	−42.16	7
61	−42.09	7
70	−42.07	6
Sitagliptin	−39.43	6
Alogliptin	−25.64	10
Vildagliptin	−5.64	6

**Table 2:** The feature set and selectivity score for each compound based on Receptor-Ligand Pharmacophore Generation.

Compounds	Number of Features	Feature Set	Selectivity Score
Comp73	5	A ^1^D ^2^N ^3^P ^4^R ^5^	11.72
Comp74	5	ADDH ^6^P	10.89
Comp55	6	AAAAHP	10.58
Comp71	6	AADHNR	10.46
Comp63	7	AADHHPR	10.05
Comp57	5	AADHP	9.64
Comp56	4	AADP	8.33
Comp54	4	AAAP	7.42
Comp72	4	ADHH	7.01
Comp65	5	ADHHR	6.94
Alogliptin	4	AAPP	8.19
Vildagliptin	4	AAHP	7.07
Sitagliptin	3	AHP	5.63

^1^ A: HB_ACCEPTOR; ^2^ D: HB_DONOR; ^3^ N: NEG_IONIZABLE; ^4^ P: POS_IONIZABLE; ^5^ R: RING_AROMATIC; and ^6^ H: HYDROPHOBIC.

**Table 3:** The total energy at 1000 ps and number of intermolecular H-bonds for each compound at different time points.

Compounds	Total Energy at 1000 ps (kcal/mol)	Different Time Points (ps)				
		200	400	600	800	1,000
Comp70	−157,714.28	11	9	7	11	12
Comp54	−156,891.72	8	6	5	5	6
Comp63	−156,672.61	12	7	8	5	7
Comp55	−156,558.42	9	14	8	11	10
Comp64	−156,449.51	6	5	5	5	5
Comp68	−156,440.91	6	5	8	4	5
Comp61	−156,331.64	8	9	10	10	6
Comp57	−156,297.55	5	10	8	9	7
Comp56	−155,533.69	5	6	10	5	6
Comp72	−155,408.55	6	7	4	8	5
Comp71	−155,240.66	3	8	6	6	6
Comp69	−155,187.79	5	5	10	9	9
Comp73	−155,187.79	5	5	10	9	9
Comp65	−154,805.59	8	7	7	6	9
Comp74	−154,805.59	8	7	7	6	9
Vildagliptin	−156,620.70	7	7	8	10	7
Alogliptin	−156,613.52	14	12	18	11	12
Sitagliptin	−156,192.52	14	17	15	14	12

**Table 4:** RMSD values of protein ligand complexes and individual ligands following MD simulation.

Average RMSD Values for Protein-Ligand Complexes	Average RMSD Values for Ligands
2P8S_Comp68 (1.44 ± 0.13 Å)	Comp68 (1.37 ± 0.20 Å)
2P8S_Comp70 (1.52 ± 0.16 Å)	Comp70 (1.66 ± 0.20 Å)
2P8S_Comp71 (1.47 ± 0.16 Å)	Comp71 (1.53 ± 0.24 Å)
2P8S_Sitagliptin (1.62 ± 0.21 Å)	Sitagliptin (1.58 ± 0.21 Å)
2P8S_Vildagliptin (1.51 ± 0.19 Å)	Vildagliptin (1.64 ± 0.26 Å)
2P8S_Alogliptin (1.58 ± 0.20 Å)	Alogliptin (1.76 ± 0.21 Å)

## Supplementary Materials

Supplementary materials can be found at http://www.mdpi.com/1422-0067/17/6/920/s1.
