# Supplementary material for: Finding a Potential Dipeptidyl Peptidase-4 (DPP-4) Inhibitor for Type-2 Diabetes Treatment Based on Molecular Docking, Pharmacophore Generation, and Molecular Dynamics Simulation
Source: Int J Mol Sci. 2016 Jun 13;17(6):920. doi: 10.3390/ijms17060920 (PMC4926453; doi:10.3390/ijms17060920)
Supplement: Supplementary file 1 [file ijms-17-00920-s001.pdf]

Supplementary Materials: Finding Potential Dipeptidyl Peptidase-4 (DPP-4) Inhibitor for Type-2 Diabetes Treatment Based on Molecular Docking, Pharmacophore Generation and Molecular Dynamics Simulation

Harika Meduru, Yeng-Tseng Wang, Jeffrey J. P. Tsai and Yu-Ching Chen

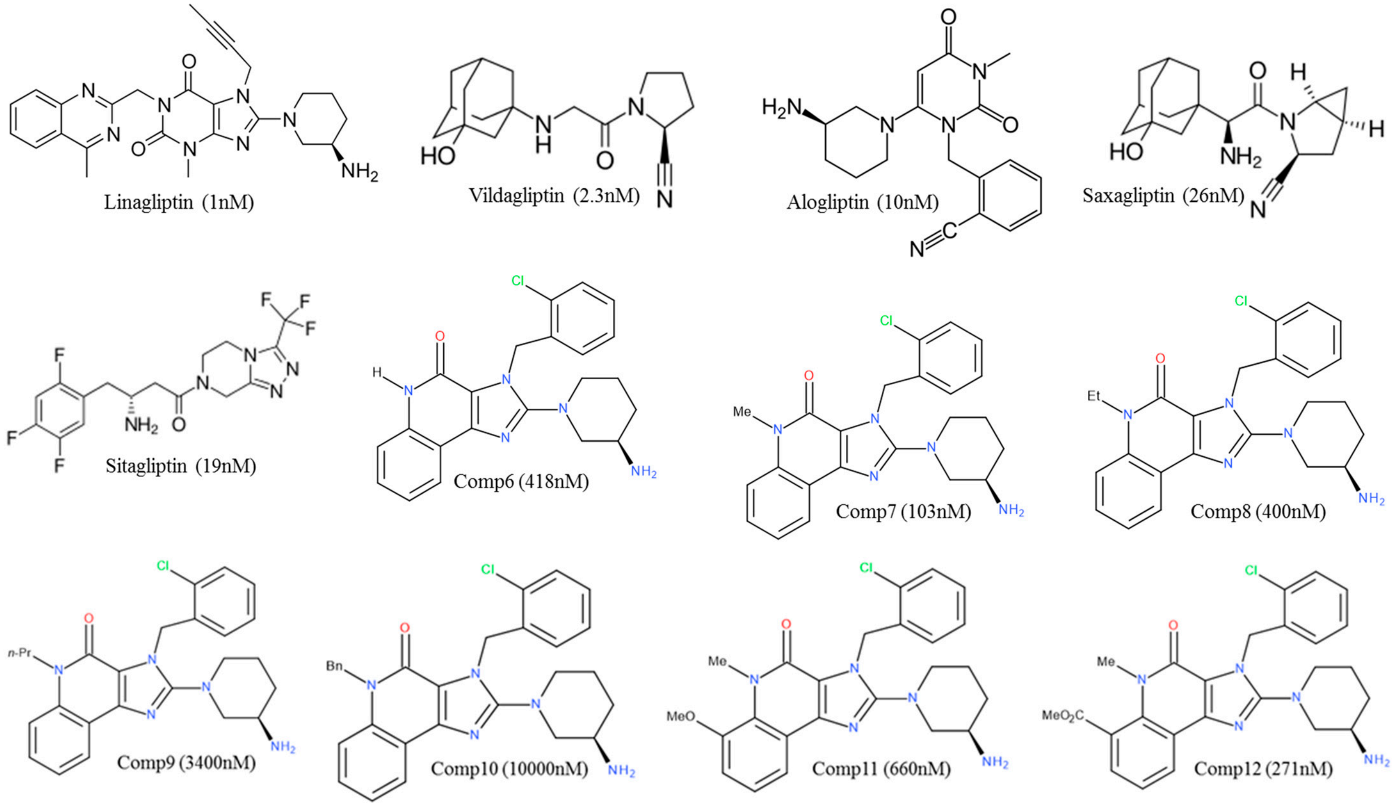

Figure S1. *Cont.*

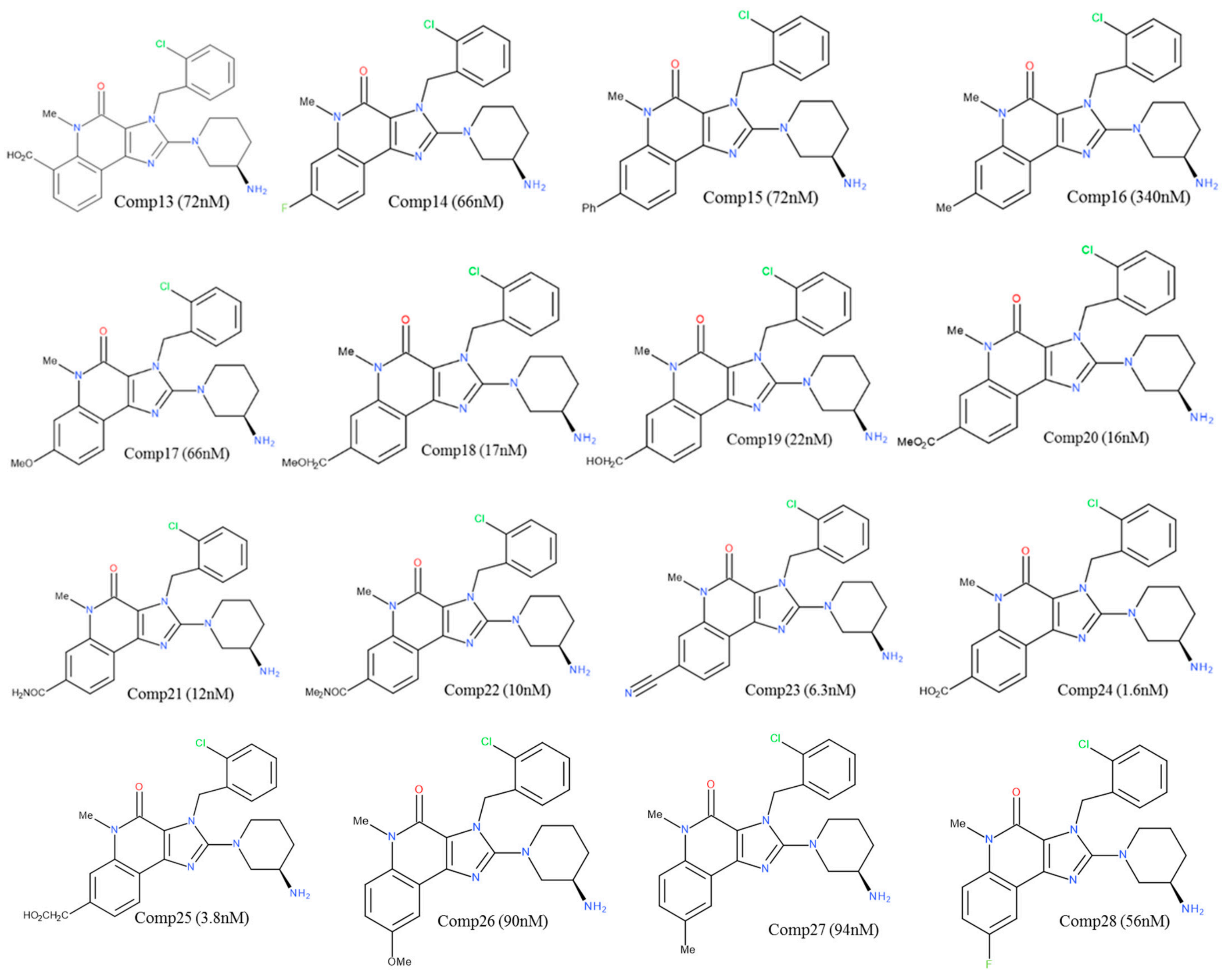

Figure S1. *Cont.*

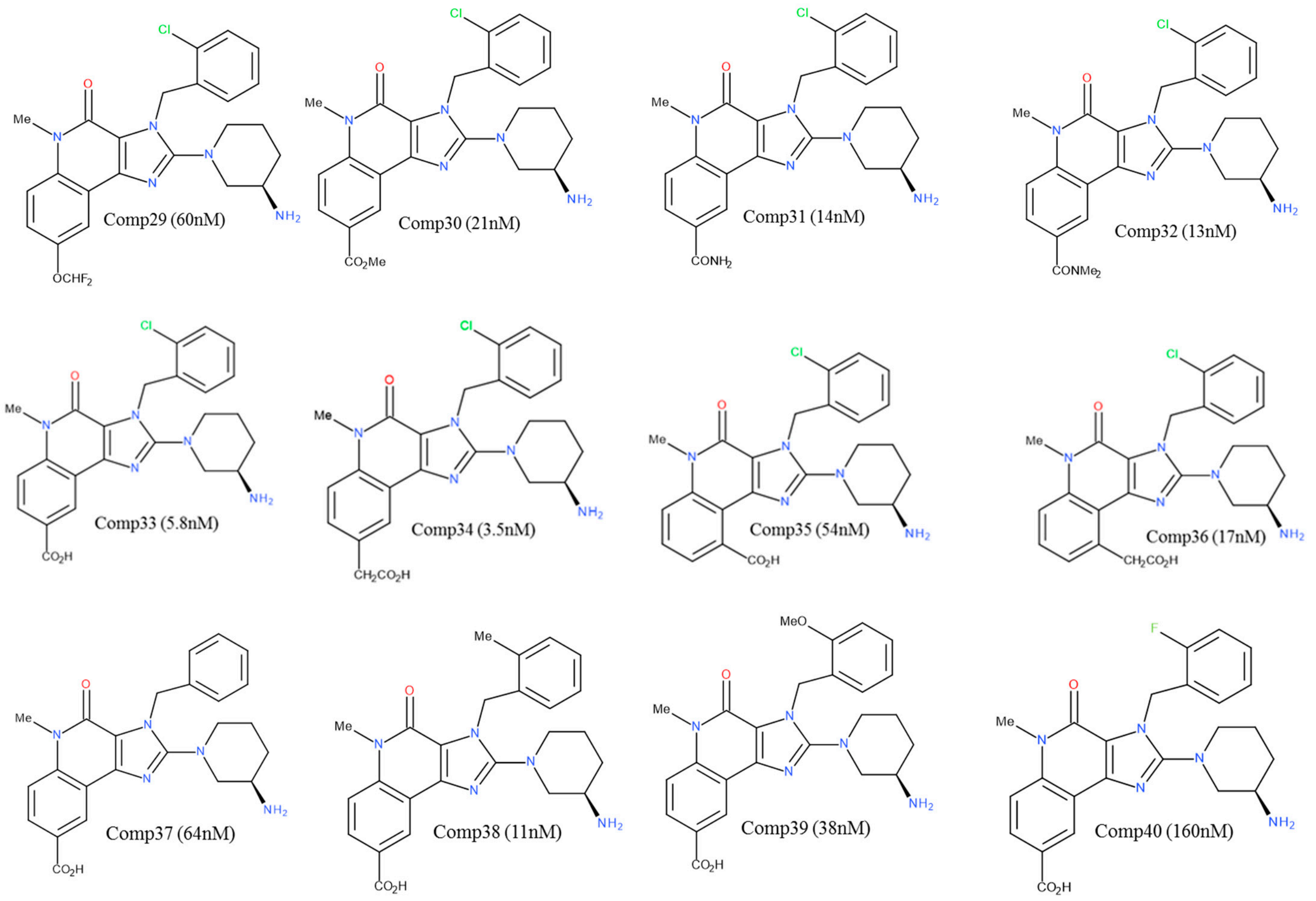

Figure S1. *Cont.*

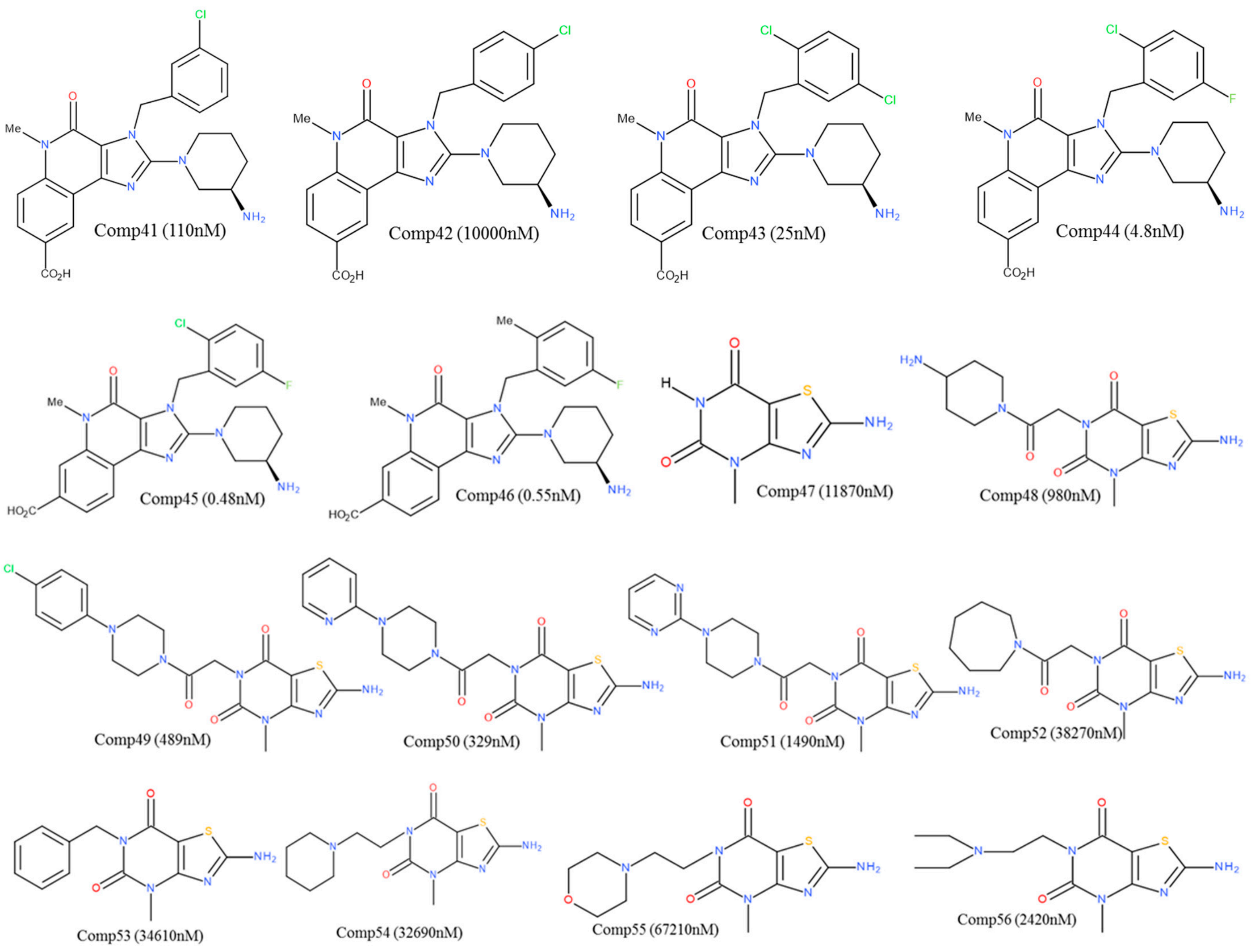

Figure S1. *Cont.*

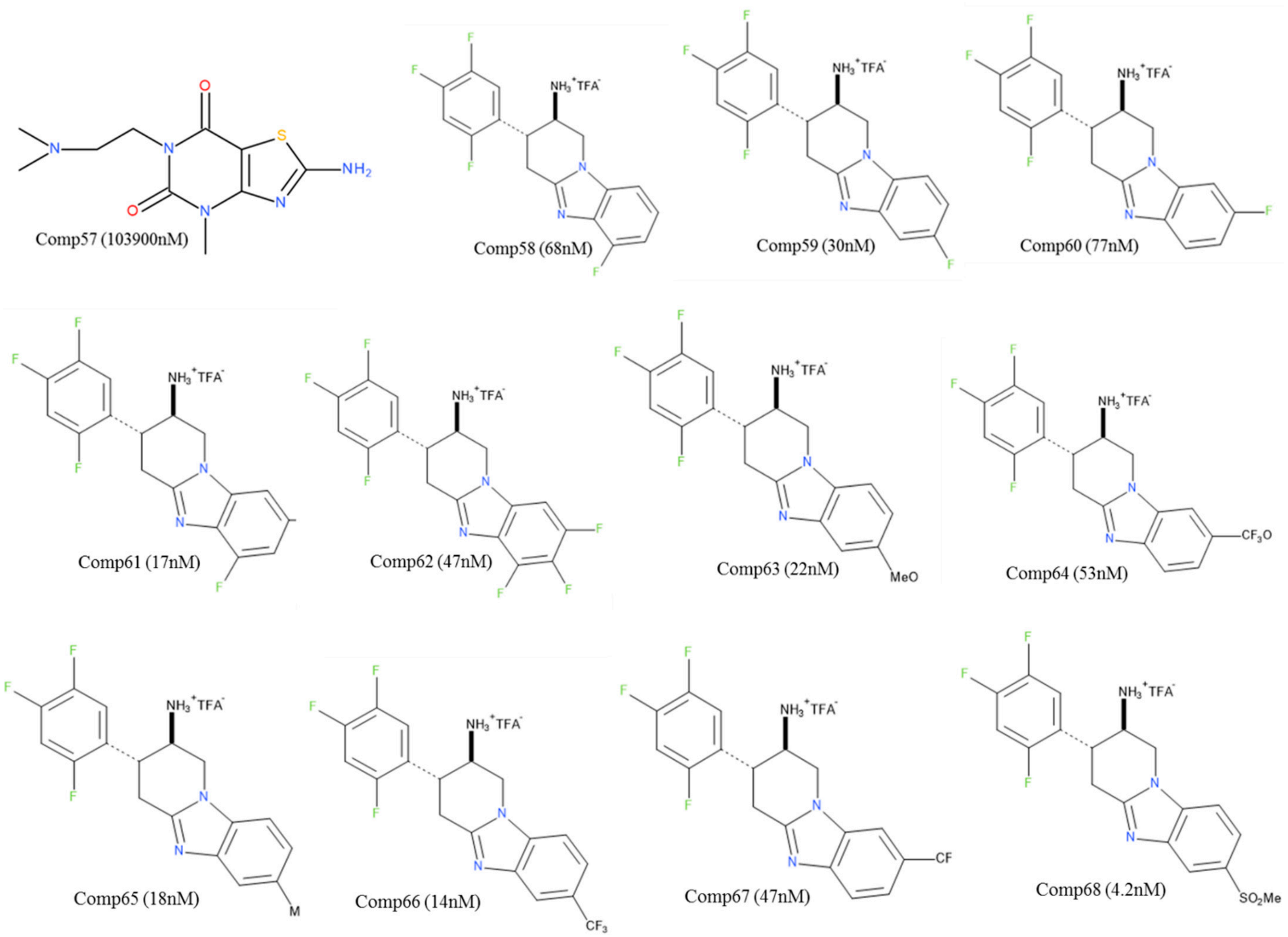

Figure S1. *Cont.*

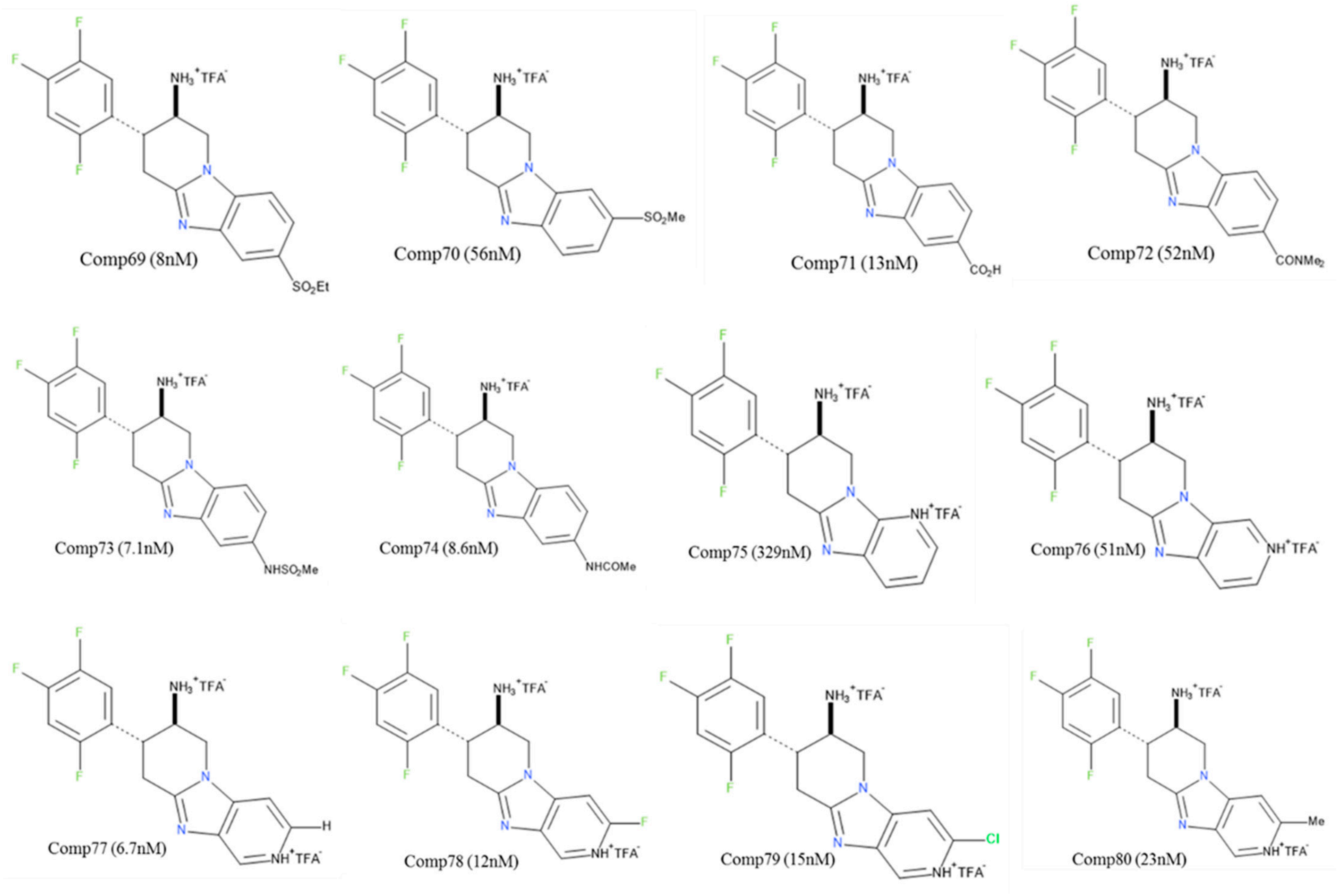

Figure S1. *Cont.*

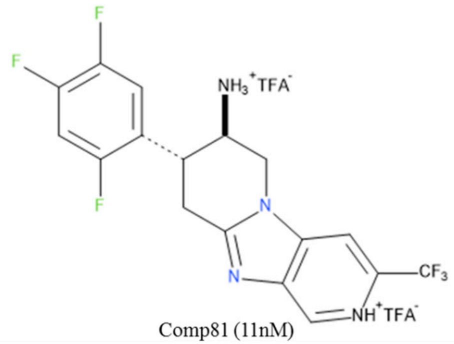

Comp81 (11nM)

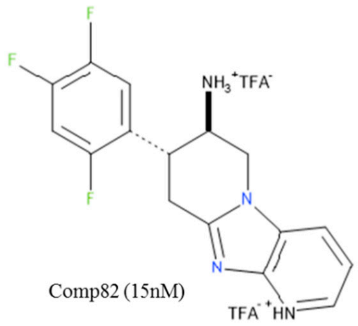

Comp82 (15nM)

Figure S1. 2D structures of 82 compounds (Aminopiperdine-fused imidazoles, Thiazolopyrimidine derivatives, quinolin-fused imidazoles) for DPP4 enzyme and the IC_{50} values are shown in quotation.
